# Acute Mesenteroaxial Volvulus in the Setting of Chronic Paraesophageal Hernia: A Case Report

**DOI:** 10.7759/cureus.34124

**Published:** 2023-01-23

**Authors:** Yu An Lin, William J Berglind, Robert Cromer

**Affiliations:** 1 General Surgery, William Carey University College of Osteopathic Medicine, Hattiesburg, USA; 2 General Surgery, Singing River Health System, Gulfport, USA

**Keywords:** case report, chronic abdominal pain, fundoplication, paraesophageal hernia, gastric volvulus

## Abstract

Hiatal hernia is a common finding in radiologic and gastroenterology evaluations. Here, we present a patient with an uncommon paraesophageal subtype who was managing her hiatal hernia symptoms conservatively and eventually developed the rare complication of mesenteroaxial gastric volvulus. The chronic history of this patient’s hiatal hernia with classic complaints suggestive of gastric ischemia prompted clinical suspicion of volvulus. Here, we discuss the initial clinical presentation of this patient, imaging study, and emergent surgical intervention by robot-assisted laparoscopic gastric volvulus reduction, hiatal hernia repair, and Nissen fundoplication. Although the size and axis of rotation of this patient’s volvulus made this case challenging, the prompt intervention prevented complications associated with volvulus and ischemia.

## Introduction

Gastric volvulus is a rare pathology that occurs when the stomach rotates on its long axis (organoaxial) or less commonly on its short axis (mesenteroaxial). The incidence of gastric volvulus peaks in the fifth decade of life and children younger than one year old comprises 10-20% of cases [1,2]. There are no apparent differences in the incidence rate among different sex or race [2]. Etiology for gastric volvulus is multifactorial; common risk factors include advanced age, obesity, pre-existing paraesophageal hernia, diaphragmatic abnormalities, phrenic nerve paralysis, and kyphoscoliosis [2,3]. In the chronic form of gastric volvulus, patients may stay asymptomatic and remain undiagnosed for years. However, the acute form of gastric volvulus often presents with symptoms of generalized, intermittent abdominal pain, nausea, and vomiting [3,4]. Prompt diagnosis and surgical intervention are required to prevent gastric ischemia, perforation, and necrosis, all of which lead to high morbidity and mortality [4]. Here, we report a case of chronic paraesophageal hiatal hernia leading to mesenteroaxial (MA) gastric volvulus and the clinical course after successful surgical intervention.

## Case presentation

A 77-year-old female with a three-year history of postprandial fullness and postprandial dull left shoulder pain presented to the emergency department (ED) with worsening postprandial discomfort, which was now accompanied by non-bilious, non-bloody vomiting. Notably, the patient underwent an esophagogastroduodenoscopy (EGD) at an outside facility approximately six months ago and was found to have a large hiatal hernia. She had no ascites, chronic constipation, or any known history of abdominal disease that would increase her risk for gastric volvulus. Given her age, history of diabetes, and chronic obstructive pulmonary disease with active tobacco use, the patient was managed non-operatively and given a proton pump inhibitor for reflux symptom control. Two weeks before her ED visit, the patient went on a cruise vacation. During the trip, she noticed that after eating or drinking anything, a sharp pain developed in the epigastric region, radiating to the left shoulder, accompanying nausea and vomiting. On initial evaluation at the cruise clinic, the patient was treated for urinary tract infection, but her gastrointestinal (GI) symptoms persisted. Her abdominal pain, nausea, and vomiting had worsened over the week and became constant, which eventually prompted her to visit the ED.

On initial ED evaluation, the patient had oxygen saturation of 94% on room air, blood pressure of 130/81 mmHg, temperature of 97.4°F, respiratory rate of 16 breaths per minute, and heart rate of 92 beats per minute. The patient was in no acute distress, but in obvious pain. She had an obese body habitus with a body mass index of 30.55, a regular heart rate and rhythm, lungs clear to auscultation bilaterally, and 3/4 pulses in all extremities. On abdominal examination, her abdomen was non-distended and had no rebound or guarding.

**Figure 1:**
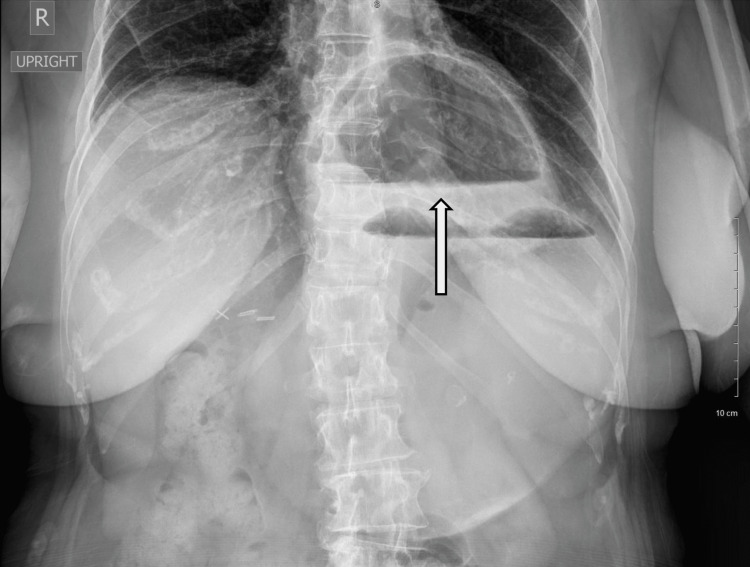
Upright radiograph Abdominal and chest radiograph in posteroanterior view shows large air-fluid level mass at the left upper quadrant, indicating a hiatal hernia (arrow). There is no sign of pneumoperitoneum, indicating no evidence of perforation.

On initial imaging, the chest posteroanterior view radiograph showed a remarkable size hiatal hernia (Figure 1).

**Figure 2:**
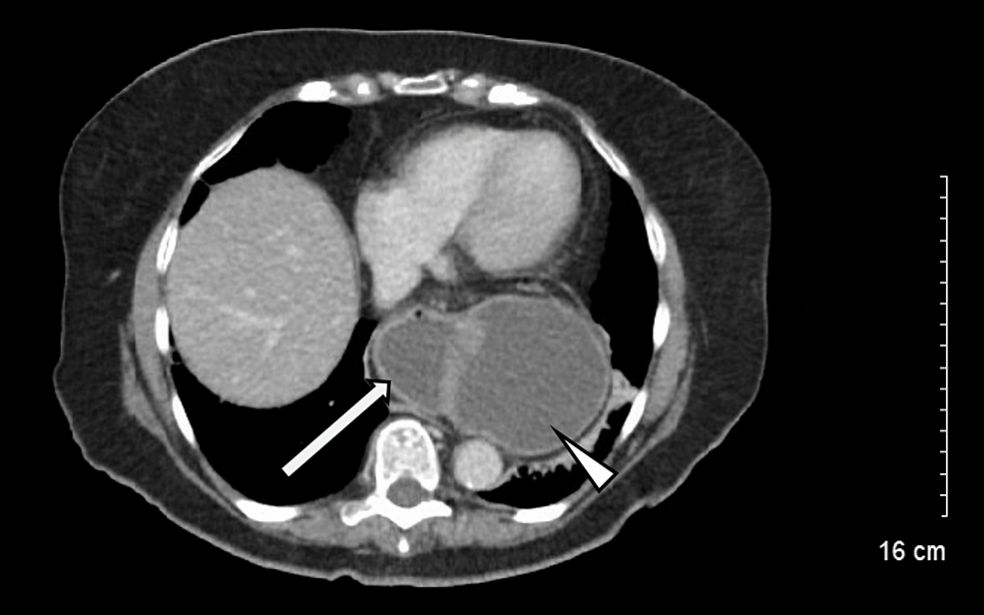
Axial computerized tomography Axial computerized tomography image shows a portion of the antrum (arrow) at the same level as fundus (arrowhead) in the mediastinum, meeting the diagnostic criteria of hiatal hernia with mesenteroaxial volvulus of the stomach. Gastric wall thickening and adjacent fluid indicate active inflammation.

Computerized tomography (CT) imaging was remarkable for a large hiatal hernia with suspected MA volvulus of the stomach; a portion of the antrum and the first portion of the duodenum were sequestered in her mediastinum (Figure 2).

A nasogastric tube (NGT) was placed, and there was a copious amount of brown/bilious appearing gastric content. The patient’s upper abdomen remained tender on palpation with associated left shoulder pain.

Due to her persistent pain after NGT placement, the patient was immediately taken to the operating room. The proximal duodenum and distal stomach appeared moderately inflamed but appeared grossly viable. Given the chronicity of this patient’s hernia, tissues within the hernia sac were very inflamed and adherent. With careful combination of blunt dissection and electrocautery, the volvulus was reduced, the hernia sac was completely excised, and the hiatal defect was closed with 0 Ethibond pledgeted sutures in an interrupted fashion. Finally, a Nissen 360-degree posterior fundoplication was performed in standard fashion using 2-0 Ethibond in an interrupted fashion over 40 French bougies. The patient tolerated the procedure well. A full liquid diet was started on postoperative day (POD) 1; full bowel function had returned by POD3. The patient had minimal discomfort and was safely discharged on POD4. At the two-week follow-up, the incision was well healed, and the patient had concerns of mild dysphasia. At the six-week follow-up, the patient recovered well without any concerns or GI symptoms. 

## Discussion

Gastric volvulus is classified based on the axis of rotation or etiology. Classifying by etiology, primary gastric volvulus is defined as volvulus due to congenital or acquired abnormalities of the gastric ligaments; it presents more commonly with chronic symptoms [3]. Secondary gastric volvulus is defined as volvulus due to anatomical abnormalities that are not associated with gastric ligamentous attachments. Instead, in secondary gastric volvulus, potential etiologies are paraesophageal hernia, phrenic nerve paralysis, and anatomic abnormalities of diaphragm or stomach [3]. In adults, paraesophageal hernia is a common cause of secondary gastric volvulus [5], like our patient in this case.

Different types of gastric volvulus classified by the axis of rotation are summarized in Table 1.

**Table 1:** Summarization of different types of gastric volvulus classified by the axis of rotation.

Type	Anatomical description	Percentage of all gastric volvulus
Type I	Stomach rotates around a long axis connecting the pylorus and the gastroesophageal junction	60%
Type II	Stomach rotates around a short axis connecting the lesser curvature to the greater curvature of the stomach	30%
Type III	Combination of Type I and II	<10%
Type IV	Unclassified	<10%

Type I is organoaxial (OA) volvulus where the stomach rotates around a long axis connecting the pylorus and the gastroesophageal junction [5]. OA volvulus is the most common type of abnormal rotation, consisting of approximately 60% of all gastric volvulus cases [6]. Type II is MA volvulus where the stomach rotates around a short axis connecting the lesser curvature to the greater curvature of the stomach. Type II encompasses approximately 30% of all gastric volvulus cases while Type III, a combination of OA and MA, and Type IV (unclassified) made up the remaining 10% [6]. Notably, MA gastric volvulus is more commonly seen in children and is rare in adults [6]. Also, paraesophageal hernia predisposes patients to OA gastric volvulus [7]. Hence, our patient’s chronic paraesophageal hernia leading to MA instead of OA gastric volvulus made her clinical diagnosis unique.

The clinical presentation of gastric volvulus can be acute, subacute, or chronic. The classical Borchardt triad of retching, severe epigastric pain, and inability to pass the NGT is present in 70% of all patients with acute gastric volvulus [8]. However, the patient in our case is the 30% of all patients who can pass the NGT but presented with two-week history of epigastric pain and retching. Our patient reflects the difficulty of making diagnosis for gastric volvulus based on clinical symptoms alone. In this case, her past medical history and clinical presentation prompted an imaging study, which ultimately aided in the diagnosis.

The initial diagnostic imaging study is typically a plain film radiograph. In the chest radiograph, gastric volvulus presents as mediastinal or retrocardiac air-fluid level. In an upright abdominal radiograph, volvulus presents as a double air-fluid level with large, distended stomach and possible collapsed small bowel [7]. When the plain film is equivocal, CT provides both high sensitivity and specificity for the diagnosis of gastric volvulus [9]. Finding of gastric antrum at the same level as or above the gastropyloric junction is diagnostic. Wall thickening, and adjacent fluid or fat stranding may also be seen. As in this case, the plain film radiograph and CT image confirmed the diagnosis of gastric volvulus.

Management and treatment of gastric volvulus start with fluid resuscitation, electrolyte correction, and NGT placement [10,11]. When an NGT cannot be placed, an immediate operation is warranted to decompress stomach and to repair hernia. When an NGT is placed, but the patient shows signs of ischemia on imaging or endoscopy study, or recurrent pain despite gastric decompression, or signs of sepsis, an immediate surgery is also warranted. The surgical management typically involves repair of the anatomic defect; however, notably, in primary volvulus or patients who are otherwise poor surgical candidates, cases have shown successful management with endoscopic de-rotation and gastropexy without hernia repair [12,13]. In our case, the patient had a successful placement of the NGT; however, she continued to have persistent abdominal pain. Given that her vitals were stable, we proceeded with robot-assisted laparoscopic hiatal hernia repair and Nissen fundoplication. Indeed, the minimally invasive approach minimized post-op recovery time and the patient had full bowel movement after three days of recovery. On POD14 follow-up, the patient tolerated diet well with mild dysphasia but without any post-op complications. At six-week follow-up, she had no concerns and was fully recovered. 

## Conclusions

Paraesophageal hernia is an uncommon subtype of gastric hernia, and acute gastric volvulus is a rarer complication of paraesophageal hernia. At presentation, acute gastric volvulus can present with vague and insidious GI symptoms such as abdominal pain, nausea, and vomiting, like our patient’s progressively worsening symptoms. These symptoms can become severe secondary to worsening gastric outlet obstruction and mucosal inflammation. Patients may present with a relatively benign physical exam with “out of proportion” subjective symptoms of pain and emesis. For this reason, having a high clinical suspicion with a low threshold for the imaging study ensures that clinicians do not miss diagnosis. While the plain film radiograph can provide evidence of gastric hernia, a CT image has a high sensitivity and specificity for making the diagnosis of gastric volvulus. Based on patient presentation after initial management, a surgeon should consider emergent surgery with hernia repair or endoscopic de-rotation with gastropexy as definitive treatment. In this case, a classic presentation of chronic paraesophageal hernia led to an uncommon complication of MA volvulus. With current treatment modalities, the patient can have good outcomes. Gastric volvulus is a diagnosis we should be vigilant for accurate and timely diagnosis to prevent further complications of ischemia and perforation.
